# Reliability of the Lateral Step-Up Test and Its Correlation with Motor Function and Activity in Chronic Stroke Survivors

**DOI:** 10.1155/2020/7859391

**Published:** 2020-04-02

**Authors:** Patrick W. H. Kwong, Shamay S. M. Ng

**Affiliations:** ^1^Department of Rehabilitation Sciences, The Hong Kong Polytechnic University, HKSAR, China; ^2^Rehabilitation Research Institute of Singapore, Nanyang Technological University, Singapore

## Abstract

**Background:**

The Lateral Step-Up Test (LSUT) has been used to evaluate the closed kinetic chain functional muscle strength in people with orthopaedic or neurological conditions. No study has systematically investigated the intrarater, interrater, and test-retest reliabilities of this measure in stroke survivors. In addition, correlations of the LSUT count with other stroke-specific impairment and function measurements remain unidentified.

**Objectives:**

This study was aimed at investigating (1) the interrater, intrarater, and test-retest reliability of the LSUT; (2) minimum detectable change in LSUT counts; and (3) correlation between LSUT counts and stroke-specific impairment and function measurements.

**Methods:**

Thirty-three stroke survivors were assessed with LSUT and a battery of stroke-specific impairment and function measurements, including Fugl-Meyer assessment of lower extremity (FMA-LE), lower limb muscle strength, Five Times Sit-to-Stand Test (FTSTS), Berg Balance Scale (BBS), Timed Up and Go Test (TUG), and Activities-specific Balance Confidence (ABC) scale, by two assessors. Their performance on LSUT was reassessed 1 week later to establish the test-retest reliability. The intraclass correlation coefficient (ICC) was used to assess the reliability of LSUT, and Spearman's rho was used to quantify the strength of correlations between LSUT counts and secondary outcomes.

**Results:**

The LSUT counts exhibited good to excellent intrarater, interrater, and test-retest reliability (ICC: 0.869–0.991). The minimum detectable change in the average LSUT count was 1 step. LSUT counts correlated significantly with the FMA-LE score, lower limb muscle strength (except for the hip abductors), FTSTS time, BBS score, TUG time, and ABC score.

**Conclusions:**

The LSUT is a reliable, valid, and easily administered measure of the closed kinetic chain functional muscle strength of stroke survivors.

## 1. Introduction

Muscle weakness is a common sequela of stroke. In survivors, the respective strengths of the paretic knee extensors and ankle dorsiflexors are typically 61% and 32% of those in the nonparetic leg [1]. Muscle strength is an important determinant of the performance of a stroke survivor. Specifically, the lower limb muscle strength, especially in the ankle dorsiflexors, is a significant predictor of walking speed and distance, as reflected in the Timed Up and Go Test (TUG) [2, 3], the 6-Minute Walk Test [2], and the Five Times Sit-To-Stand Test (FTSTS) [4]. Clinically, muscle strength is usually assessed manually or with a handheld dynamometer [5]. However, these assessments measure the open kinetic chain muscle strength and thus cannot simulate the actions used in daily life. In contrast, closed kinetic chain assessments are more similar to functional movements and therefore may provide greater insights into functional neuromuscular control in stroke survivors [6].

The Lateral Step-Up Test (LSUT) is a closed kinetic chain test used to assess functional muscle strength of the lower limbs [7, 8]. This test was designed to assess concentric and eccentric lower limb muscle strength, as well as balance and proprioceptive sense [7]. To date, the LSUT has been used as a clinical measure of functional muscle strength in healthy adults [7, 9], older adults with hip fracture [8], patients with knee meniscectomy [10], and patients with cerebral palsy [11]. This measure has demonstrated excellent test-retest reliability (intraclass correlation coefficients (ICC) = 0.85–0.92) in elderly patients after hip fracture [8]. In a study of adolescents with cerebral palsy, the LSUT also exhibited moderate to high correlations with TUG and FTSTS scores and the time required to ascend and descend stairs [11]. Although a functional muscle strength assessment is a critical component of stroke rehabilitation, the interrater, intrarater, and test-retest reliability of the LSUT and the correlations between this measure and stroke- related impairments have not been investigated in stroke survivors.

Accordingly, this study was aimed at investigating the following parameters in stroke survivors: (1) the interrater, intrarater, and test-retest reliability of the LSUT counts; (2) the minimum detectable change (MDC) in the LSUT counts on the paretic and nonparetic sides; and (3) the correlations of LSUT counts with the Lower Extremity subscale of the Fugl-Meyer Assessment (FMA-LE) score, lower limb muscle strength, FTSTS completion time, Berg Balance Scale (BBS) score, TUG completion time, and Activities-specific Balance Confidence (ABC) score.

## 2. Methods

### 2.1. Participants

The protocol for this cross-sectional study was approved by the institutional ethics committee. The people with stroke were informed in advance about the objectives and procedures of the study and provided signed informed consent. All procedures involving human participants followed the guidelines set by the Declaration of Helsinki.

A power analysis indicated that a sample of 27 people with stroke and the collection of 2 observations per people with stroke would be required to detect an ICC of 0.90 at a confidence level of 0.05 and power of 81% [12]. Accordingly, 33 stroke survivors (22 men, 11 women; mean age: 60.18 ± 6.42 years) were recruited from a local self-help group for stroke survivors via convenience sampling. The following inclusion criteria were applied: (i) age ≥ 50 years, (ii) an interval of at least 1 year between the stroke event and study enrollment, and (iii) the ability to stand up from a chair independently. People with stroke were excluded if they (i) were unable to follow verbal instructions, (ii) received a score < 6 on the Chinese version of the Abbreviated Mental Test (AMT) [13], (iii) were medically unstable, or (iv) had a comorbid neurological or musculoskeletal condition.

### 2.2. Assessment Procedure

**Figure 1:**
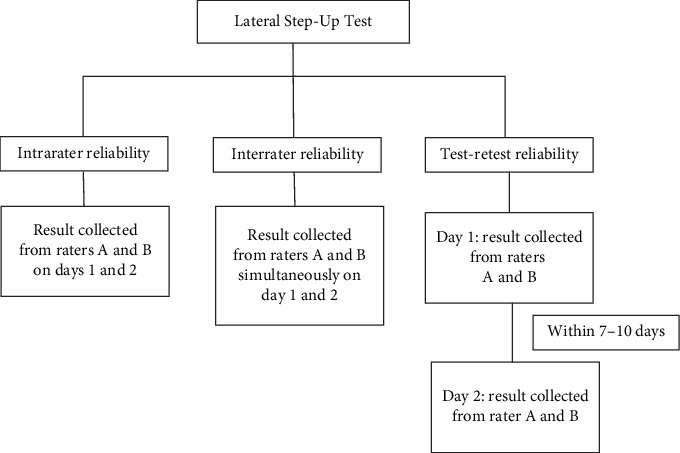
Data collection procedures.

All people with stroke were required to attend two assessment sessions separated by a 1-week interval, during which they were assessed independently by two raters (Figure 1). The people with stroke also completed muscle strength tests, the FTSTS, and the BBS and FMA-LE scales in a random order. A resting period of five minutes was offered between each assessment. All the raters were final year physiotherapy students who received additional training in conducting the assessments.

### 2.3. Outcomes

#### 2.3.1. Lateral Step-Up Test (LSUT)

The LSUT was administered using a 15 cm step [7]. People with stroke were asked to stand with their feet shoulder width apart and in a parallel position and to place the foot of the leg to be tested on the step. Subsequently, the people with stroke were given the following standardized verbal instruction: “When I count to 3, fully straighten the leg on the step and then bend the knee again until the other foot touches the floor. Perform as many repetitions as possible in 15 seconds.”. After a practice trial to ensure their understanding of the test, each participant performed two trials on each side. People with stroke could wear AFO during the test while walking aids were not allowed.

#### 2.3.2. Lower Extremity Subscale of the Fugl-Meyer Assessment (FMA-LE)

The FMA-LE measures coordination, reflexes, and voluntary movement [14] and is widely accepted as a reliable assessment for stroke survivors (ICC: 0.83–0.95) [15]. This subscale comprises 17 items scored on a scale of 0–2, yielding a maximum total possible score of 34. A higher FMA-LE score suggests a lesser degree of lower extremity impairment on the paretic side.

#### 2.3.3. Lower Limb Muscle Strength

The maximum voluntary isometric contractions of the hip abductors, knee flexors and extensors, and ankle dorsiflexors and plantarflexors were measured bilaterally using a Nicholas handheld dynamometer (model 01,160; Lafayette Instrument Company, Lafayette, IN, USA) in accordance with a standardized assessment protocol [16]. This type of measurement has been shown to assess muscle strength accurately, with good to excellent reliability (ICC: 0.77–0.97) [17]. Three trials of each muscle were performed on each side during each session, and each trial was separated by a 1-minute rest period. The averages of the three trials were used in the analyses.

#### 2.3.4. Five Times Sit-to-Stand Test (FTSTS)

The FTSTS is a highly reliable (ICC = 0.99) measure used to evaluate the functional performance and lower limb muscle strength of chronic stroke survivors [4]. The time required to rise from a standardized chair with a 45 cm high seat without armrests and sit down again five times as quickly as possible was recorded. After a practice trial, two times were recorded for each participant during each session, and the average was used in further analyses.

#### 2.3.5. Berg Balance Scale (BBS)

The BBS is a reliable tool (ICC: 0.98–0.99) used to assess the static and dynamic balance of people with balance impairments [18]. The assessment involves 14 functional tasks scored on a scale from 0 to 4, for a maximum total possible score of 56. A higher BBS score suggests a better balance ability.

#### 2.3.6. Timed Up and Go Test (TUG)

Functional mobility was measured using the TUG, which was previously shown to yield excellent test-retest reliability (ICC = 0.95) in people with chronic stroke [2]. Participants were allowed to use walking aids if needed. The times required to complete three trials were measured with a stopwatch, and the average time was used in further analyses.

#### 2.3.7. Activities-Specific Balance Confidence (ABC)

The ABC scale assesses a person's self-perceived ability to maintain balance while performing daily functional activities (i.e., subjective balance confidence). The Chinese version of the ABC scale was used in this study [19]. This scale contains 16 items scored from 0 to 100, and a higher ABC score suggests better subjective balance confidence. This scale was shown to have good test-retest reliability (ICC = 0.85) in people with chronic stroke [20].

### 2.4. Statistical Analysis

SPSS software (version 23.0; IBM Corp., Armonk, NY, USA) was used for the data analysis. Descriptive statistics were compiled by summarizing the participants' demographic data. Intrarater reliability was analyzed using ICC_3,1_ which correlated the LSUT counts of the two trials on each side measured by different examiners and on different days. Interrater reliability was examined using ICC_3,2_, which correlated the mean LSUT counts for the two trials on each side between the two examiners on different days. Test-retest reliability was established using ICC_3,2_, which correlated the mean LSUT counts of the two trials on each side with different examiners between the two assessments.

Only the participants' body mass index values were normally distributed. Wilcoxon's signed-rank test was used to estimate the significance of the observed differences in LSUT counts between the paretic and nonparetic sides of the people with stroke. Because the sample size was relatively small, Spearman's correlation coefficients (*r*_s_) were used to assess the relationships between the LSUT and other outcome measures. The strength of each correlation was estimated using the obtained *r*_s_, such that *r*_s_ values of <0.25, 0.25–0.50, 0.50–0.74, and >0.75 indicate little or no, fair, moderate to good, and good to excellent correlations, respectively [21]. Moreover, the minimum detectable change (MDC) was generated using the following formula:

(1) $$\begin{array}{c} \text{MDC}=1.96\times \text{SEM}\times \sqrt{2}, \end{array}$$

where SEM is the standard error of the measurement of the LSUT counts and was calculated as

(2) $$\begin{array}{c} \text{SEM}=S\sqrt{1-\rho }. \end{array}$$

Here, *S* is the standard deviation of the LSUT counts and *ρ* is the ICC for test-retest reliability. For all measures, a *p* value ≤ 0.05 was considered to indicate statistical significance.

## 3. Results

The participants' demographics are summarized in Table 1. The average poststroke duration of the participants was 9.35 years. A previous study reported that an FMA-LE score of 21 could be used to classify the level of mobility in chronic stroke survivors [22]. Based on the results, 12 people with stroke were considered to have poor mobility function and 21 of them demonstrated a high level of mobility function in the current study. There was no significant difference in the average LSUT counts between the paretic and nonparetic legs (*p* = 0.255). The LSUT results demonstrated excellent intrarater (ICC = 0.936 and 0.983) and interrater (ICC = 0.979 and 0.991) reliability and good to excellent test-retest reliability (ICC: 0.869–0.903) for the paretic and nonparetic legs (Table 2). The estimated MDC values of the average step counts were <1 step (0.45 and 0.49 step on the paretic and nonparetic sides, respectively).

Spearman's correlation analysis showed significant correlations between the average step counts on both the paretic and nonparetic sides and the FMA-LE scores (paretic: *r*_s_ = 0.511, nonparetic: *r*_s_ = 0.535), knee flexor strength (paretic: *r*_s_ = 0.371, nonparetic: *r*_s_ = 0.405), knee extensor strength (paretic: *r*_s_ = 0.437, nonparetic: *r*_s_ = 0.436), ankle dorsiflexor strength (paretic: *r*_s_ = 0.376, nonparetic: *r*_s_ = 0.364), plantarflexor strength (paretic: *r*_s_ = 0.507, nonparetic: *r*_s_ = 0.507), FTSTS completion time (paretic: *r*_s_ = −0.477, nonparetic: *r*_s_ = −0.544), TUG completion time (paretic: *r*_s_ = −0.397, nonparetic: *r*_s_ = −0.438), BBS score (paretic: *r*_s_ = 0.561, nonparetic: *r*_s_ = 0.585), and ABC score (paretic: *r*_s_ = 0.444, nonparetic: *r*_s_ = 0.441) (Table 3).

## 4. Discussion

This is the first published study to investigate the intrarater, interrater, and test-retest reliability of LSUT counts in a sample of people with chronic stroke. Consistent with previous studies of young, healthy adults [7] and adolescent (12–18 years) patients with cerebral palsy [11], our data indicated excellent intrarater, interrater, and test-retest reliability for the LSUT counts. These values are, of course, contingent on the use of standardized instructions and equipment. Thus, our standardized instructions, which were provided by two groups of examiners, may have maximized the observed interrater reliability. In addition, the two examination days were separated by a 1-week interval to minimize any learning effects and avoid fatigue.

Surprisingly, this study revealed no significant differences between the average LSUT counts of the paretic and nonparetic legs. It is possible that the motor control and muscle strength of the limb on the ipsilesional side are affected in people with stroke [23, 24]. Moreover, several compensatory strategies were observed during the testing of the paretic leg. First, the paretic knee was braced in hyperextension while standing, and movement was completed via contralateral hip hiking and ipsilateral trunk side-flexion which lifted the nonparetic leg. When testing the nonparetic leg, most of the people with stroke hesitated to land on the paretic leg, possibly due to reduced ankle proprioception after stroke [25].

The LSUT counts on both the paretic and nonparetic legs exhibited significant positive correlations with the FMA-LE scores, a measure of stroke-specific motor impairment [14]. This correlation may indicate the effects of stroke-specific impairments and the need for paretic side coordination during testing of the nonparetic side. Similarly, the LSUT counts on both sides exhibited significant positive correlations with the strengths of all lower limb muscles on the paretic side, except for the hip abductors. During the LSUT, activation of the dorsiflexors on the tested leg shifts the center of mass (COM) upward and forward to initiate stepping up, while coactivation of the dorsiflexors and plantarflexors enhances ankle stability for balance control [26, 27]. The hip abductors on the tested leg work simultaneously to keep the pelvis level throughout the single-leg stance phase [28]. Concentric and eccentric contractions of the knee extensors and flexors, respectively, were then performed to fully extend the knee in a controlled manner. A return to the starting position requires the eccentric contraction of the knee extensors, which shifts the COM downwards in a controlled manner. The LSUT is in effect a test of functional muscle strength. Therefore, the counts would be expected to correlate significantly and positively with the lower limb muscle strengths.

Surprisingly, the paretic side LSUT counts and hip abductor strengths did not correlate significantly in this study, although this outcome may have been due to the limited sample size. Nevertheless, this result may also have been affected by compensatory movements, as changes in the effects of gravity can cause differences in measured muscle strengths among testing positions. The observed differences in the correlations of various muscle strengths with LSUT counts may be due to the closed chain concentric and eccentric demands of muscles in the LSUT, as the maximum isometric hip abductor strength was assessed in an open chain position.

Both the paretic and nonparetic LSUT counts correlated significantly with multiple functional assessments, including the BBS score, FTSTS completion time, and TUG completion time. The LSUT involves rapid COM movements and single-leg standing, which greatly challenge balance and proprioception. Items 12 (placing alternate feet on a stool) and 14 (standing on one foot) of the BBS evaluation also involve single-leg standing, which would explain the significant correlation. The FTSTS was designed to measure functional muscle strength in the lower extremities [29] in a closed chain position. In this sense, it is similar to the LSUT, which is highly specific for the lower limb muscles in the weight-bearing leg that lift the body up and down in a closed chain position [30]. Moreover, the significant negative correlation between the two LSUT counts and the TUG completion time was consistent with the findings of a previous study of adolescents with cerebral palsy, although the correlation was weaker in the current study. This discrepancy may be due to interstudy differences in disease pathophysiology, age distribution, sample size, and spasticity.

Moreover, the LSUT counts exhibited a fair yet significant positive correlation with the ABC score, a measure of balance confidence during daily functional activities [19]. The ABC is a real world-based measurement, and therefore, its ratings can easily be affected by factors such as the patient's past experiences, fear of falling, and a general lack of self-confidence in addition to their actual physical functions [31]. Therefore, the nature of the ABC score may explain the observed correlation.

## 5. Limitations

This study had several limitations. First, the measured time was given priority, whereas the quality of movement was not considered during testing. Second, various factors that would be expected to affect performance, such as spasticity, movement plan, lower limb proprioception, and tactile sensation, were not assessed. Third, the standardized step height was not optimal for all participants because of variations in leg length and height. Fourth, all of the people with stroke had relatively good levels of mobility; namely, 54% walked unaided and 42% used a cane. Therefore, this study may have been subject to selection bias, and the results should not be generalized to all stroke survivors. Fifth, the sample size was selected based on reliability (the main study objective) and may not have been sufficiently large to detect significant correlations between the LSUT counts and other outcome measures. Further studies with larger samples are clearly warranted.

## 6. Conclusions

The LSUT yielded excellent intrarater, interrater, and test-retest reliability when applied to a sample of chronic stroke survivors. Moreover, the bilateral LSUT counts correlated significantly and positively with the FMA-LE scores, lower limb muscle strength (except for the hip abductors), FTSTS time, BBS score, TUG time, and ABC score. In conclusion, the LSUT is a reliable, effective, and easy-to-administer clinical assessment of functional muscle strength in stroke survivors.

## Figures and Tables

**Table 1:** Demographics of the stroke survivors (*n* = 33).

Characteristics	Mean ± SD
Age (year)	60.18 ± 6.42
Height (cm)	161.29 ± 7.03
Weight (kg)	66.81 ± 12.21
Body mass index (kg m^−2^)	25.50 ± 3.20
Poststroke period (years)	9.35 ± 4.28
No. of falls in the past 6 months	0.42 ± 0.79
AMT	9.36 ± 0.86
Average LSUT count (steps)	
Paretic side	8.86 ± 3.24
Nonparetic side	8.58 ± 2.84
	*n*
Gender (male/female)	22/11
Paretic side (left/right)	9/24
Aids employed (unaided/stick/frame)	18/14/1

AMT: Abbreviated Mental Test; LSUT: Lateral Step-Up Test.

**Table 2:** Reliability of the LSUT with stroke survivors.

	Rater	Day	Side	Mean LSUT count ± SD	ICC (95% CI)
Intrarater reliability-ICC_1,3_	1	1	Paretic	8.24 ± 0.46	0.954 (0.909–0.977)
			Nonparetic	8.41 ± 0.52	0.936 (0.873–0.968)
		2	Paretic	8.98 ± 0.57	0.963 (0.873–0.968)
			Nonparetic	9.33 ± 0.65	0.983 (0.966–0.992)
	2	1	Paretic	8.12 ± 0.48	0.949 (0.895–0.975)
			Nonparetic	8.24 ± 0.53	0.946 (0.892–0.973)
		2	Paretic	8.97 ± 0.55	0.941 (0.885–0.970)
			Nonparetic	9.44 ± 0.65	0.969 (0.938–0.984)

Interrater reliability-ICC_2,3_	1-2	1	Paretic	8.18 ± 0.47	0.991 (0.982–0.996)
			Nonparetic	8.32 ± 0.52	0.979 (0.957–0.989)
		2	Paretic	8.98 ± 0.56	0.989 (0.978–0.995)
			Nonparetic	9.39 ± 0.64	0.984 (0.968–0.992)

Test-retest reliability-ICC_2,3_	1	1-2	Paretic	8.61 ± 0.49	0.888 (0.758–0.946)
			Nonparetic	8.87 ± 0.56	0.869 (0.718–0.937)
	2	1-2	Paretic	8.54 ± 0.50	0.893 (0.681–0.955)
			Nonparetic	8.84 ± 0.57	0.903 (0.760–0.956)

ICC: intraclass correlation coefficient; CI: confidence interval.

**Table 3:** Outcome measures and correlations between LSUT counts and other outcome measures.

Parameters	Outcomes (mean ± SD)	Correlation coefficient with paretic LSUT counts	*p*	Correlation coefficient with nonparetic LSUT counts	*p*
FMA-LE	23.82 ± 6.23	0.511	0.002^∗∗^	0.535	0.001^∗∗^
Muscle strength					
Hip abductors					
Paretic	14.00 ± 4.37	0.333	0.058	0.318	0.071
Nonparetic	16.40 ± 3.74	0.289	0.103	0.263	0.139
Knee flexors					
Paretic	9.72 ± 7.77	0.371	0.033^∗^	0.405	0.019^∗^
Nonparetic	22.61 ± 10.16	0.004	0.981	0.020	0.913
Knee extensors					
Paretic	27.09 ± 18.11	0.437	0.011^∗^	0.436	0.011^∗^
Nonparetic	46.52 ± 21.41	0.07	0.700	0.108	0.550
Ankle dorsiflexors					
Paretic	9.49 ± 5.47	0.376	0.031^∗^	0.364	0.037^∗^
Nonparetic	15.15 ± 3.25	0.398	0.022^∗^	0.399	0.021^∗^
Ankle plantarflexors					
Paretic	13.87 ± 5.66	0.507	0.003^∗∗^	0.507	0.003^∗∗^
Nonparetic	18.78 ± 4.26	0.48	0.789	0.095	0.600
FTSTS	19.24 ± 9.96	-0.477	0.005^∗∗^	-0.544	0.001^∗∗^
TUG	15.90 ± 6.26	-0.397	0.022^∗^	-0.438	-0.011^∗^
BBS	48.60 ± 4.44	0.561	0.001^∗∗^	0.585	<0.001^∗∗^
ABC	77.93 ± 15.60	0.444	0.010^∗∗^	0.441	0.010^∗^

^∗^ *p* ≤ 0.05, ^∗∗^*p* ≤ 0.01. ABC: Activities-specific Balance Confidence scale; BBS: Berg Balance Scale; FMA-LE: Fugl-Meyer assessment of lower extremity; FTSTS: Five Times Sit-to-Stand Test; LSUT: Lateral Step-Up Test; TUG: Timed Up and Go Test.

## Data Availability

The data used to support the findings of this study are available from the corresponding author upon request.
